# Microfluidically frequency-reconfigurable compact self-quadruplexing tunable antenna with high isolation based on substrate integrated waveguide

**DOI:** 10.1038/s41598-024-51645-z

**Published:** 2024-01-09

**Authors:** Rusan Kumar Barik, Slawomir Koziel

**Affiliations:** 1 Engineering Optimization and Modeling Center, Reykjavik University, 102 Reykjavík, Iceland; 2 Faculty of Electronics, Telecommunications and Informatics, Gdansk University of Technology, 80-233 Gdańsk, Poland

**Keywords:** Engineering, Electrical and electronic engineering

## Abstract

This communication presents a novel concept of microfluidically frequency-reconfigurable self-quadruplexing tunable antenna for quad-band applications. At the initial design stage, a substrate-integrated square cavity is divided into four unequal quarter-mode cavity resonators by inserting an X-shaped slot on the top surface of the cavity. Applying four 50-Ω microstrip feed-lines to these four quarter-mode cavity resonators enables quad-band operation with self-quadruplexing capabilities. The feed lines are organized orthogonally and off-center, which leads to port isolation greater than 32.3 dB. An equivalent network model is developed to validate the proposed antenna. To realize frequency reconfigurability, two microfluidic channels corresponding to each port are created by engraving the bottom surface of the cavity. To create a reconfigurable self-quadruplexing antenna, the channels are either filled with air or dielectric liquids of higher permittivity, so that the design offers independent tunability of the operating frequencies. As a proof of concept, the prototype of a self-quadruplexing tunable antenna is fabricated and validated through measurements. The antenna prototype occupies a footprint area of 0.37λ_g_^2^. The design exhibits frequency tuning ranges of 350 MHz (8.3%), 500 MHz (10.3%), 610 MHz (11.2%), and 845 MHz (14.1%) for the first, second, third, and fourth operating bands, respectively. In all bands and across the entire tuning range, the realized gains of the designed antenna exceed 4.05 dBi. The electromagnetic modeling responses agree extremely well with the measured characteristics.

## Introduction

The innovation of multi-purpose antennas has been driven by the recent growth in the diversity of wireless communication systems and their applications. The substrate-integrated waveguide (SIW) technology has become a prominent choice in the design of such antennas due to the high-quality factor, small footprint, high gain, superior radiating properties, and ease of integration with planar devices^1–4^. In Ref.^2^, investigation on quarter-mode SIW (QMSIW) and its application to antenna implementation has been studied. This QMSIW-based antenna has a low footprint, high gain, and excellent radiation characteristics. A multi–input–multi–output (MIMO) antenna array has been realized for 5G mm-wave applications^3^. An antenna array has been implemented based on SIW and a graphene layer for sub-terahertz applications^4^. The above antennas are single-fed and single-/wide-band characteristics and therefore are not feasible for multiband applications. Thus, growing demand for multi-functional antennas has led to devising several SIW architectures, employed to realize dual-, triple-, quad-, and penta-band devices^5–9^. For instance, in Ref.^5^, a dual-band antenna array has been designed by using a quarter-mode SIW cavity. A triple-band dual-polarized antenna has been realized based on a SIW square cavity^6^. In Ref.^7^, a quad-band planar inverted-F antenna has been designed using U-shaped slots. The probe-fed metal plate has been used to develop planar triple-band and quad-band antennas^8^. An omnidirectional antenna has been implemented based on a square columnar structure and three radiating slots^9^. The above-mentioned filtering and multi-band antennas have single-feed lines, which require frequency-selective devices in order to choose the resonating frequency. Also, these antennas exhibit poor isolation.

To address the aforementioned issues, a number of SIW-based self-diplexing^10–14^, self-triplexing^15^, self-quadruplexing^16–24^, self-pentaplexing^25^, and self-hexaplexing^26,27^ antennas have been studied. A U-shaped slot has been engraved on the top-surface of the SIW to construct a self-diplexing antenna^10^. A compact self-diplexing antenna has been realized based on a bowtie-ring slot backed by SIW^11^. Based on shielded QMSIW, an extremely compact self-diplexing antenna has been presented^12^. To create a tunable antenna, a half-mode SIW cavity filled with a varactor diode-loaded slot has been used^13^. To design a tunable self-diplexing antenna, SIW-based modified A-shaped slot with microfluidic channels have been employed^14^. To create a self-triplexing antenna, a shielded half-mode SIW loaded with an inverted U-shaped slot has been used^15^. The use of cavity-backed four V-shaped slots has led to the development of a self-quadruplexing antenna^16^. Circular polarized self-quadruplexing antennas have been designed using SIW square cavity-backed four T-shaped slots^17^. A miniature self-quadruplexing antenna has been built using a circular SIW cavity filled with C-shaped and arc-shaped slots^18^. Four radiating patches have developed on the topside of the SIW cavity to produce a self-quadruplexing antenna^19^. Using quarter-mode SIW cavities filled with arc-shaped slots, a miniature self-quadruplexing antenna has been realized^20^. A self-quadruplexing antenna for microwave and mm-wave applications has been created by converting a SIW square cavity into two half-mode cavities^21^. Based on SIW cavity-backed four separately-fed U-shaped slots, a self-quadruplexing multiple-input multiple-output (MIMO) antenna has been developed^22^. In Ref.^23^, an ultra-compact self-quadruplexing antenna has been realized based on U-shaped slots. In Ref.^24^, half-mode SIW cavity resonators have been employed for the realization of a compact SQA foe microwave and mm-wave applications. Utilization of the SIW cavity featuring T-shaped and П-shaped slots, led to a development of a self-quintuplexing antenna^25^. SIW cavity-backed six radiating patches have been used to create a self-multiplexing antenna for hexa-band applications^26^. A self-hexaplexing antenna has been constructed using a substrate-integrated rectangular cavity loaded with two П-shaped slots^27^. The self-multiplexing antennas such as the ones listed above feature fixed resonating frequencies. The aforementioned structures must be modified in order to access new operating frequencies, which will increase the cost of the system while incurring additional time expenditures. Investigating options for design of frequency-reconfigurable self-multiplexing antennas is a challenging endeavor, yet necessary to solve the aforementioned issues.

Due to their advantages over micro-electromechanical systems (MEMS) and semiconductor devices, microfluidically frequency reconfigurable microwave passive components have recently attracted considerable attention^28–32^. Employing a liquid–metal switching mechanism, a frequency-reconfigurable microstrip antenna has been developed^28^. The use of capacitive-microfluidic switches has been used to create a frequency-configurable microstrip slot antenna^29^. Fluidic channels have been used to produce a frequency-tunable annular slot antenna with a coplanar waveguide-feed^30^. Fluidic channels have been used to produce a dual-band microstrip annular slot antenna that is independently controllable^31^. A tunable monopole antenna with a capacitive coupled mechanism produced by substrate stack-up has been implemented using a microfluidic channel^32^.

This article introduces a unique concept for a self-quadruplexing tunable antenna that can be microfluidically frequency-configurable for quad-band applications. First, an X-shaped slot is inserted on the top surface of a square substrate-integrated cavity to divide it into four unequal quarter-mode cavity resonators (QMCR). These resonators are excited by 50-Ω microstrip feed-lines to obtain quad-band operation with self-quadruplexing capability. Two microfluidic channels that correspond to each QMCR are constructed by carving the cavity's bottom surface in order to enable frequency-reconfigurability. The adjustment of operating frequencies is realized by filling the channels with air or distilled water. For validation, a prototype of the microfluidic frequency-reconfigurable self-quadruplexing tunable antenna is constructed and experimentally demonstrated. The original contributions of the paper and the key advantages of the antenna presented in this study are as follows:

1. To the best of the author's knowledge, a microfluidically frequency-reconfigurable self-quadruplexing tunable antenna is reported for the first time in the open literature;
2. The presented antenna has a small footprint area of 0.37λ^2^;
3. Verification of the proposed frequency adjustable antenna has been carried out using an equivalent circuit model;
4. Frequency tunability is straightforward and only requires filling two fluidic channels associated at each QMCR with various liquids of high relative permittivity;
5. The antenna exhibits frequency tuning ranges of 350 MHz (8.3%), 500 MHz (10.3%), 610 MHz (11.21%), and 845 MHz (14.1%) for the first, second, third, and fourth operational bands, respectively;
6. The minimum isolation level is greater than 32.3 dB at all bands and across the entire tuning range;
7. The realized gain of the antenna is better than 4.05 dBi in all bands and over the entire tuning range.

## Methods

### Antenna design and analysis

**Figure 1:**
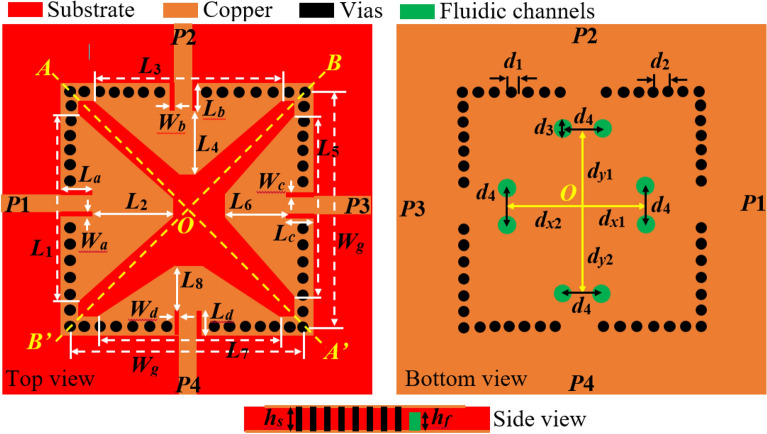
Layout of the proposed microfluidically frequency tunable SIW-based self-quadruplexing antenna. The geometry parameter values are: *W*_*g*_ = 30.0, *L*_1_ = 23.0, *L*_2_ = 11.328, *L*_3_ = 23.0, *L*_4_ = 9.249, *L*_5_ = 23.0, *L*_6_ = 7.889, *L*_7_ = 23.0, *L*_8_ = 6.4889, *L*_*a*_ = 3.1711, *W*_*a*_ = 0.245, *L*_*b*_ = 2.75111, *W*_*b*_ = 0.245, *L*_*c*_ = 2.6111, *W*_*c*_ = 0.215, *L*_*d*_ = 2.51111, *W*_*d*_ = 0.211, *d*_1_ = 1.0, *d*_2_ = 2.0, *d*_3_ = 1.6, *d*_4_ = 3.0, *d*_*x*1_ = 8.0, *d*_*x*2_ = 10.0, *d*_*y*1_ = 9.0, *d*_*y*2_ = 10.5; unit: mm.

**Figure 2:**
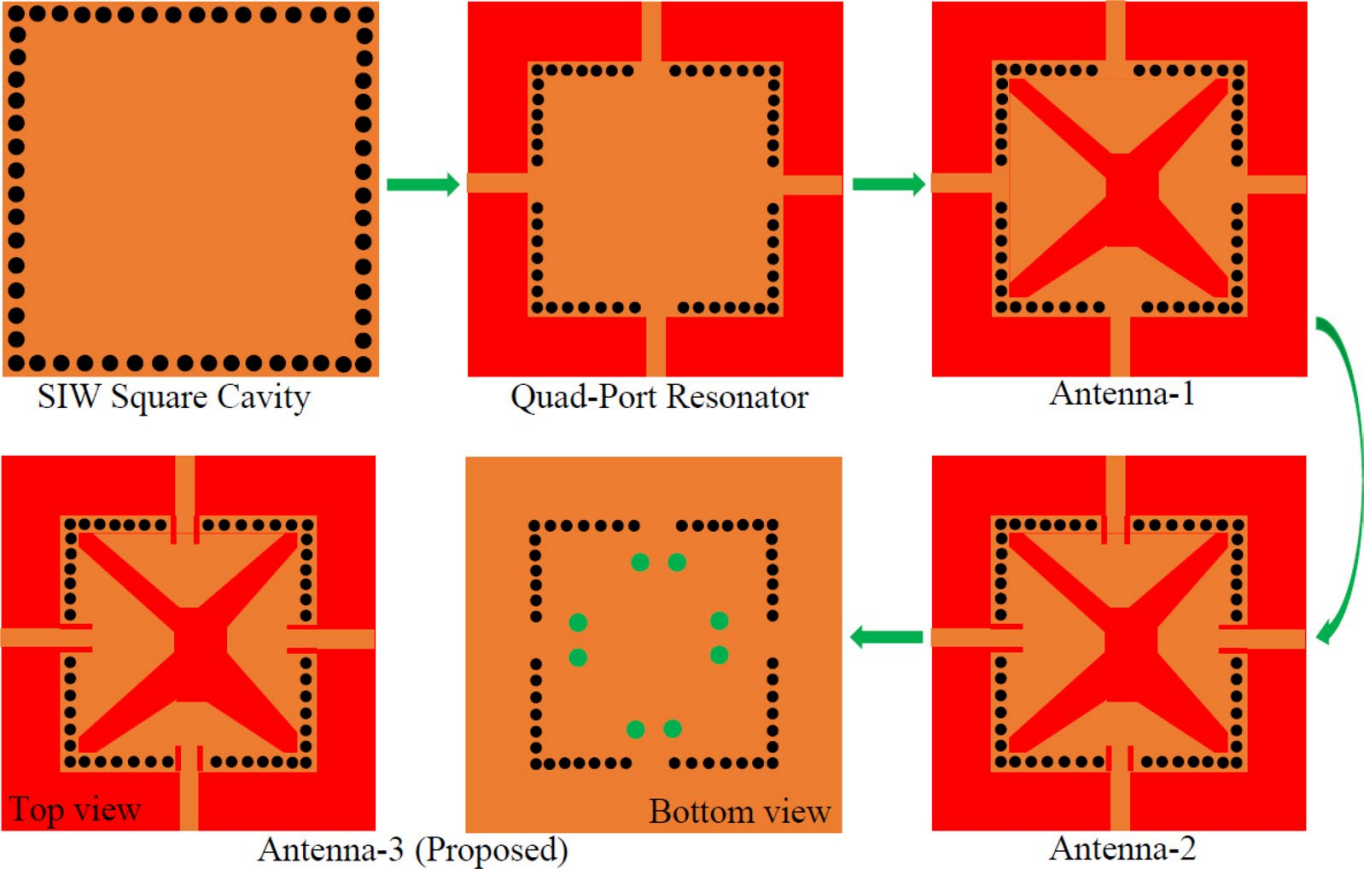
A simple step-by-step development of tunable antenna design.

The geometry of the proposed frequency tunable self-quadruplexing antenna with optimized dimensions has been depicted in Fig. 1. The CST Microwave Studio is used to simulate and analyze the proposed tunable antenna. The antenna is constructed using a square cavity, an X-shaped slot, four microstrip feed lines, and fluidic channels. Figure 2 shows the design stages of the device. Initially, a SIW square cavity (30 mm × 30 mm) is designed to operate in TE_110_ mode at 4.55 GHz and TE_120_/TE_210_ modes at 7.20 GHz. The mode frequencies of the proposed substrate-integrated square cavity are determined as^1^:

1 $$f_{mn0}^{SISC} = \frac{1}{{2\sqrt {\mu \varepsilon } }}\sqrt {\left( {\frac{m\pi }{{W_{{_{eff} }}^{SISC} }}} \right)^{2} + \left( {\frac{n\pi }{{L_{{_{eff} }}^{SISC} }}} \right)^{2} }$$

where m = 1, 2,…, n = 1, 2,…, and *ε* = *ε*_0_*ε*_*r*_, *µ* = *µ*_0_*µ*_*r*_ are the permittivity and permeability of the substrate. The effective width $W_{{_{eff} }}^{SISC}$ and length $L_{{_{eff} }}^{SISC}$ are^1^:

2 $$\left\{ \begin{gathered} W_{{_{eff} }}^{SISC} = W_{g} - 1.08\frac{{d_{1}^{2} }}{{d_{2} }} + 0.1\frac{{d_{1}^{2} }}{{W_{g} }} \hfill \\ L_{{_{eff} }}^{SISC} = L_{g} - 1.08\frac{{d_{1}^{2} }}{{d_{2} }} + 0.1\frac{{d_{1}^{2} }}{{L_{g} }} \hfill \\ \end{gathered} \right.$$

**Figure 3:**
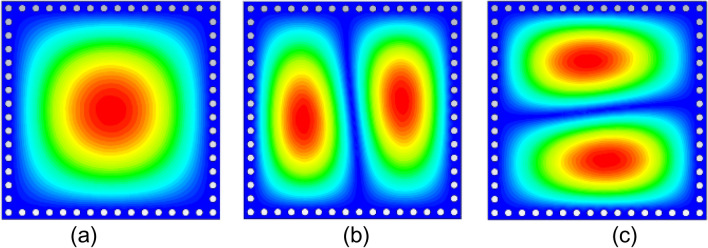
E-field distributions of conventional SIW square cavity resonator. (**a**) *TE*_110_ mode, (**b**) *TE*_210_ mode, and (**c**) *TE*_120_ mode.

**Figure 4:**
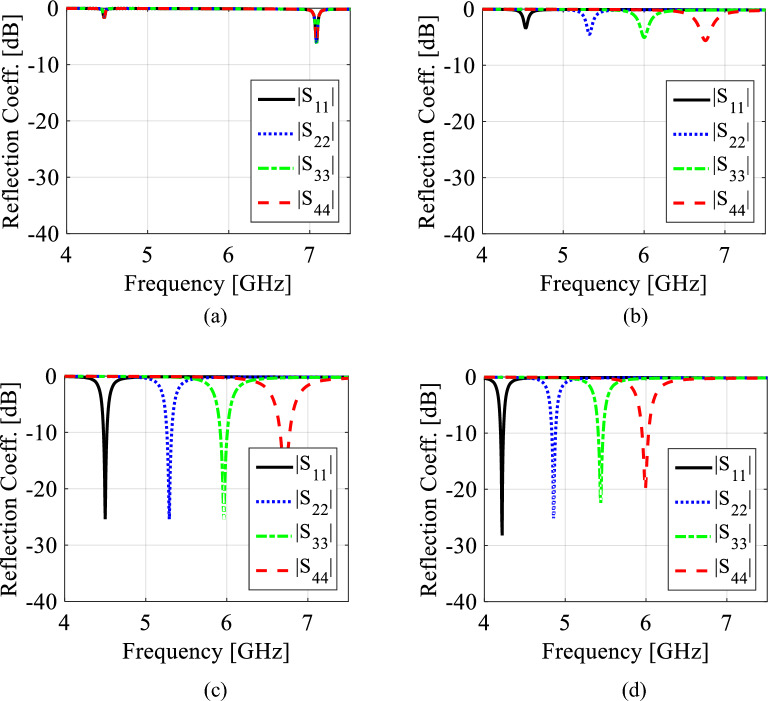
EM simulated reflection coefficients for various design stages. (**a**) Quad-port resonator, (**b**) Antenna-1, (**c**) Antenna-2, and (**d**) Antenna-3 (proposed).

**Figure 5:**
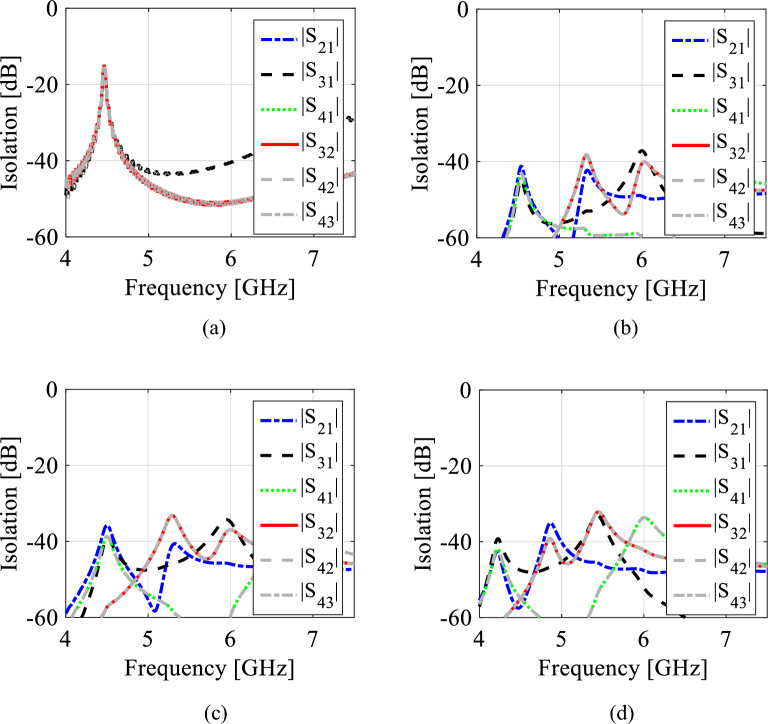
EM simulated transmission coefficients for various design stages. (**a**) Quad-port resonator, (**b**) Antenna-1, (**c**) Antenna-2, and (**d**) Antenna-3 (proposed).

The diameter *d*_1_ and center-to center distance *d*_2_ of the metallic vias are chosen using the relationship as *d*_1_/*λ* ≤ 0.1 and *d*_1_/*d*_2_ ≥ 2, in order to avoid energy leakage from the cavity^1^. Next, an X-shaped slot is engraved on the top plane to generate four distinct quarter-mode cavity resonators (QMCR) for radiation at four different operating frequencies. The spacing between the operating frequencies is depends on the dimensions of the quarter-mode cavity resonators. Therefore, we can obtain suitable operating frequencies based on our requirement using the dimensions of QMCR. Figure 3 shows the electric field distributions of TE_110_ and TE_120_/TE_210_ modes of the square cavity, which is obtained by employing the eigenmode solver. It is found that the maximum field strength for TE_110_ mode is concentrated at the center of the square cavity, whereas the maximum field strength for TE_120_/TE_210_ modes is found near the sides of the cavity. Subsequently, a quad-port resonator is developed by attaching four 50Ω feed lines to the four sides of the square cavity. The reflection and transmission coefficients at each port of the quad-port resonator are depicted in Figs. 4a and 5a, respectively. Referring to Fig. 4a, small dips in reflection coefficients are observed at 4.46 GHz and 7.08 GHz with very poor matching. As seen in Fig. 5a, the resonator exhibits a reasonable isolation between the ports since the ports are placed in orthogonal to each other. Next, an X-shaped slot is carved on the top plane of the square cavity which creates four distinct quarter-mode cavity resonators (QMCR) associated with each port. As a result, the QMCRs generate four different radiating frequencies. The reflection and transmission coefficients at each port are shown in Figs. 4b and 5b, respectively. It can be observed that at four distinct frequencies, four corresponding reflection coefficients are produced for the respective ports. Each port exhibits isolation better than 37 dB as depicted in Fig. 5c. As a result, Antenna-1 is capable of operating at four frequencies with inherent self-quadruplexing capabilities. Referring to Fig. 4b, the reflection coefficient level is high due to the impedance mismatch between the resonators and the feed lines. Therefore, Antenna-2 is developed by inserting inline feeding at each port to improve impedance matching. The S-parameters of the Antenna-2 are depicted in Fig. 4c. It can be seen that the reflection coefficients at each port are better than − 25 dB. The isolation of Antenna-2 is decreased to 33.2 dB due to the existence of a small cross-coupling path between the ports. Finally, Antenna-3 is realized by creating two fluidic channels at each QMCR to enable frequency tunability. The fluidic channels are filled with various dielectric liquids to obtain frequency tunability. For example, as Figs. 4d and 5d illustrate, the S-parameters are obtained when the fluidic channels are filled with distilled water. It can be observed that the operating frequencies are shifted to lower values since distilled water has a high relative permittivity.

### Operating principle

**Figure 6:**
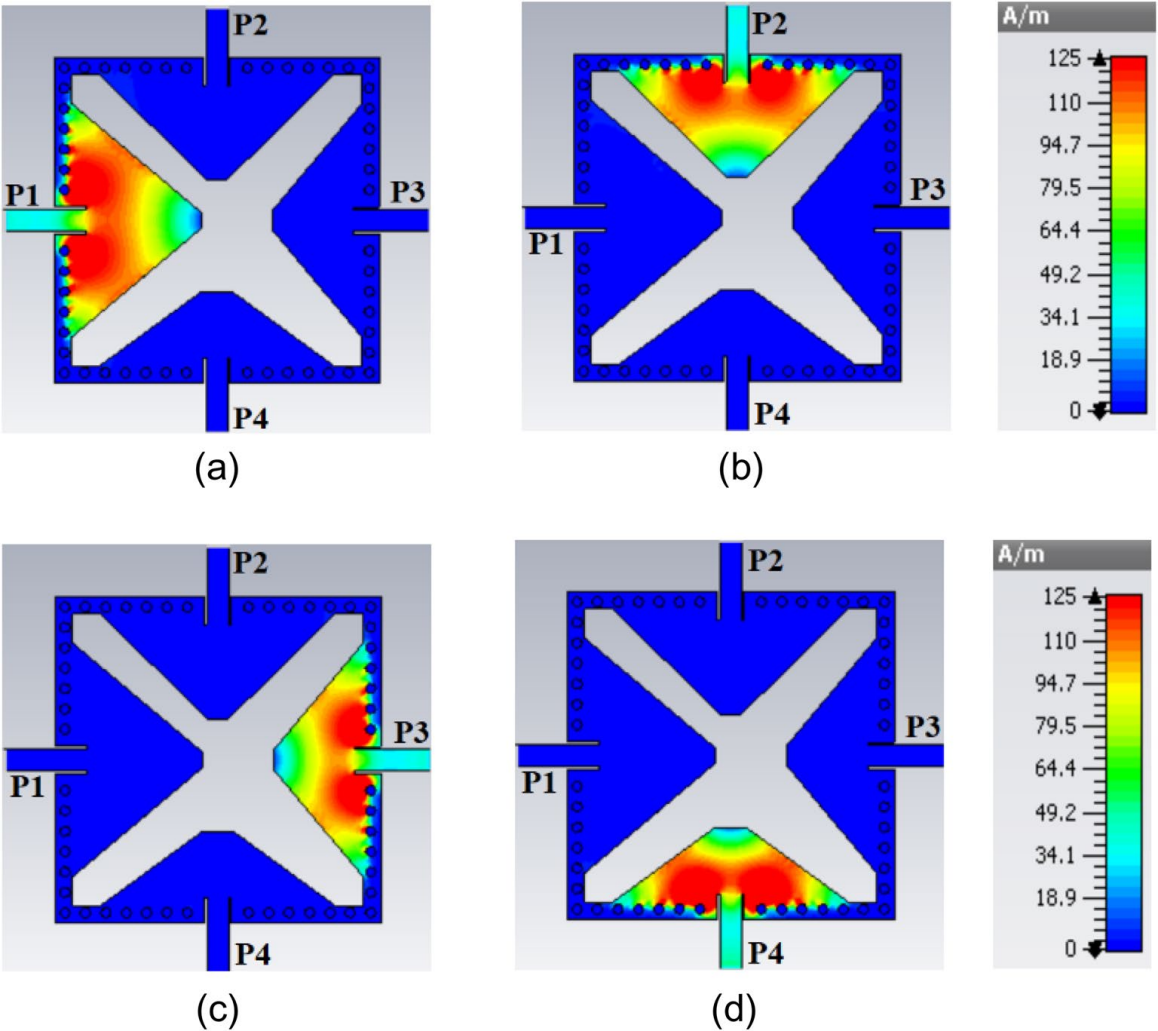
Surface current density at individual ports: (**a**) Port-1, (**b**) Port-2, (**c**) Port-3, and (**d**) Port-4.

Initially, a SIW square cavity with the dimension of 0.65*λ*_*g*_ × 0.65*λ*_*g*_ is realized to operate in the TE_110_ mode at 4.56 GHz and TE_120_/TE_210_ modes at 7.21 GHz. Then an X-shaped slot is engraved on the top plane to generate four distinct quarter-mode cavity resonators (QMCR). Referring to Fig. 1, the triangular-shaped QMCRs are defined as AOB′, AOB, BOA′, and B′OA′ resonators. Each resonator is stimulated by using an independent 50Ω feed line to enable radiation at a designated frequency. As a result, four radiating frequencies are obtained based on the four QMCRs. Figure 6 depicts the results of using an eigenmode solution to calculate surface current densities for the purpose of better comprehending the radiation mechanism. To determine these surface current densities, an excitation signal is applied to one port, whereas the remaining ports are terminated with 50Ω loads. As seen in Fig. 6, one port experiences the largest current flow, while the current flow through the remaining ports is negligible. It is evident from Fig. 6a that when Port 1 is stimulated, the AOB′ resonator radiates at 4.5 GHz for the application to 5G fixed wireless access (FWA). When Port 2 is activated, as shown in Fig. 6b, the AOB resonator radiates at 5.35 GHz for public safety band (PSB) applications. As seen in Fig. 6c, the BOA′ resonator radiates at 5.97 GHz for wireless local area network (WLAN) application when excitation is applied at Port 3. Similarly, the B′OA′ resonator operates at 6.75 GHz for INSAT or extended C-band applications when Port 4 is activated, as shown in Fig. 6d. Thus, the proposed antenna generates the self-quadruplexing capability as well as quad-band operation. To ensure good radiation features, the inline feed technique is used to establish impedance matching between the QMCRs and the feeding lines. Each port has an isolation level of at least 32.3 dB because of the orthogonal port arrangement and the X-shaped slot, which create a very weak cross-coupling path between the ports. Finally, a completely mechanical passive tuning technique utilizing fluidic channels is applied to tune a single frequency separately or the entire working frequency at once. To enable frequency adjustment, high relative permittivity liquids are poured into the fluidic channels.

### Circuit model analysis

**Figure 7:**
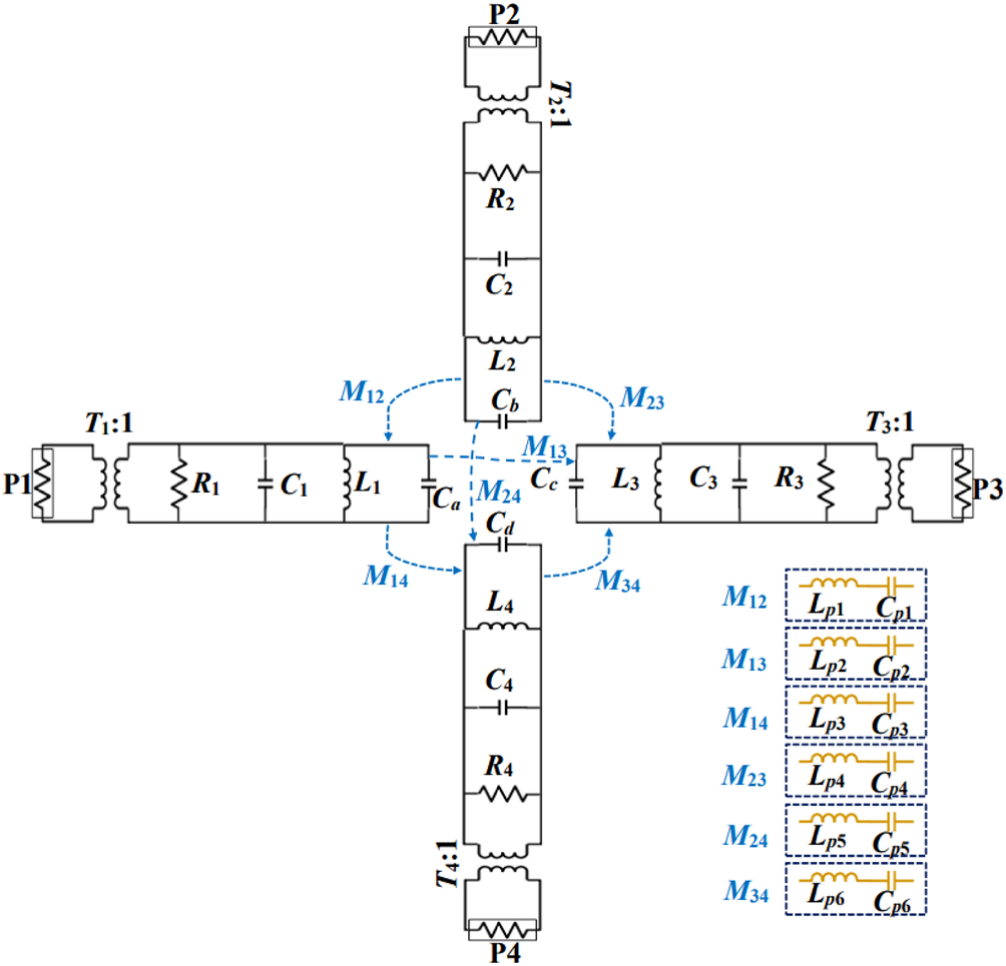
Equivalent circuit model of the proposed antenna.

To further understand the working concept of the proposed antenna, an equivalent circuit model has been assembled and shown in Fig. 7. Each QMCR resonator is modelled as a parallel RLC (*R*_*i*_, *L*_*i*_, and *C*_*i*_) network. The position of the feed lines associated with the QMCRs is responsible for the resistance *R*_*i*_, here *i* = 1, 2, 3, and 4, stands for ports 1, 2, 3, and 4, respectively. The length and width of the QMCRs are expressed by the equivalent capacitance *C*_*i*_ and inductance *L*_*i*_. The length and the width of the X-shaped slot are responsible for loading of an extra capacitance to each QMCR, which is modeled by the capacitance *C*_*a-d*_. The dimensions of the inline feeding associated with each port are responsible for the impedance matching between the QMCRs and the ports, which are modeled by the impedance transformers *T*_*i*_. The gap between the QMCR resonators is responsible for the mutual couplings (*M*_12_, *M*_13_, *M*_14_, *M*_23_, *M*_24_, and *M*_34_). The couplings are represented by series inductance *L*_*p*_ and capacitance *C*_*p*_. The resonant frequencies and input impedances associated with each QMCR resonators are calculated as^23,25^:

3 $$f_{ii} = \frac{1}{{2\pi \sqrt {L_{i} C_{a - d} } }}$$

4 $$Z_{input} = \frac{{jw_{ii} L_{i} }}{{1 - w_{ii}^{2} C_{a - d} L_{i} }}$$

**Figure 8:**
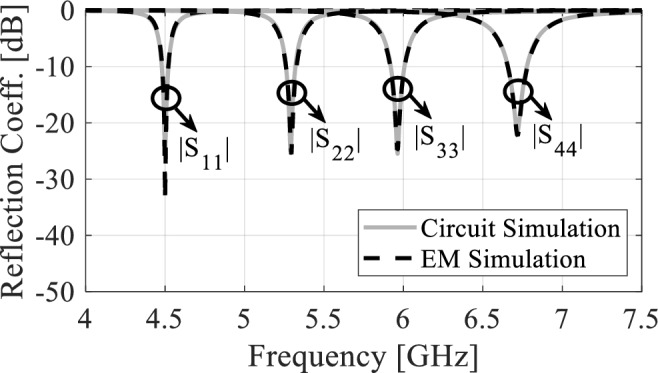
Circuit and EM simulation reflection coefficients.

Equation (3) shows that the excess capacitance *C*_*a-d*_ and inductance *L*_*i*_ have an impact on the resonating frequency. The extra capacitances are affected by the dimension of the X-shaped slot. Therefore, the resonant frequency can be altered by adjusting the slot size. Furthermore, as the inductance Li depends on the QMCRs' dimensions, any change in those dimensions will result in a frequency shift. Keysight Advanced System Design is used to validate the circuit model, and the results are compared to the EM simulation as shown in Fig. 8. Table 1 displays the values of the circuit model components.

**Table 1:** Component values of the circuit model.

*R*_1_	*L*_1_	*C*_1_	*C*_*a*_	*L*_*p*1_	*C*_*p*1_	*L*_*p*5_	*C*_*p*5_
435	0.1716	0.01	7.2895	12.0	4.507	11.243	4.6569
*R*_2_	*L*_2_	*C*_2_	*C*_*b*_	*L*_*p*2_	*C*_*p*2_	*L*_*p*6_	*C*_*p*6_
181	0.1119	0.0593	8.0739	12.914	5.0406	12.0	4.507
*R*_3_	*L*_3_	*C*_3_	*C*_*c*_	*L*_*p*3_	*C*_*p*3_	*T*_1_	*T*_2_
415	0.2511	0.02	2.8399	12.91	12.451	0.339	0.499
*R*_4_	*L*_4_	*C*_4_	*C*_*d*_	*L*_*p*4_	*C*_*p*4_	*T*_3_	*T*_4_
397	0.2611	0.0155	2.1483	15.0	4.507	0.329	0.329

### Frequency tunability

Frequency reconfigurability is achieved by applying a microfluidic channel-based fully mechanical passive tunable method. Depending on the needs, the resonant frequencies can be tuned concurrently or individually. To facilitate tunability, two fluidic cylindrical channels are carved within the bottom plane of each QMCR resonator. The diameter and height of each cylindrical via are 1.6 mm and 0.562 mm, respectively. To protect the radiating apertures, the height of the channels is smaller than the substrate height. The fluidic channels are filled with ethyl acetate (*ε*_*r*_ = 6.02), acetone (*ε*_*r*_ = 20.2), and distilled water (*ε*_*r*_ = 78.4) to enable frequency tunability. The loading of dielectric liquids altered the total relative permittivity of the substrate, resulting a shift in the resonant frequency. Two options are investigated for tunability. In the first case, just one fluidic channel is filled with dielectric liquids. In the second case, two fluidic channels are filled with the liquids.

**Figure 9:**
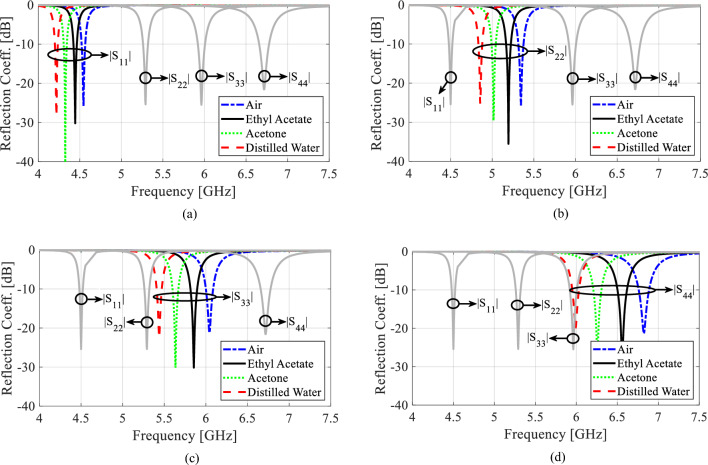
Tunability of individual resonating frequency associated with each port for various dielectric liquids. (**a**) Port-1, (**b**) Port-2, (**c**) Port-3, and (**d**) Port-4.

Figure 9 demonstrates the individual operating frequency tunability when two fluidic channels are filled with dielectric liquids. As shown in Fig. 9a, the first resonant frequency associated with Port 1 can be altered from 4.22 GHz to 4.57 GHz (a tuning range of 8.29% or 330 MHz). The percentage change in the operating frequency at each port is calculated by following (5):

5 $$FTR_{i} \left( \% \right) = \frac{{f_{i}^{\max } - f_{i}^{\min } }}{{f_{i}^{\min } }} \times 100$$

where, *FTR*_*i*_ represents the frequency tuning range in percentage, *f*_*i*_^max^ and *f*_*i*_^min^ represent the maximum and minimum frequencies changes when a port is excited and the channels are filled with the air and distilled water, respectively, here i = 1, 2, 3 and 4 stands for the port indices. It can be seen from Fig. 9b that the second operating frequency provides a 500 MHz or 10.3% tuning range (from 4.85 to 5.35 GHz). As seen in Fig. 9c, the third operational frequency associated with Port 3 may be varied between 5.44 and 6.05 GHz (a tuning range of 610 MHz or 11.21%). Finally, Fig. 9d demonstrates that the tuning range of 14.1% or 840 MHz (from 5.99 to 6.83 GHz) is possible with the fourth resonating frequency, which corresponds to Port 4. Table 2 shows the tunability of individual operating frequencies considering both scenarios. The green-colored circles in Table 2 indicate the fluidic channel filled with dielectric liquids, while the blue-colored circle indicates the fluidic channel filled with an AD250 substrate. However, the volume and relative permittivity of various dielectric liquids may be used to adjust the tuning range of the operating frequencies.

**Table 2:** Tunability of resonating frequency of the proposed antenna.

Port excitation	None (air filled)		Ethyl acetate		Acetone		Distilled water	
							
Port 1 (GHz)	4.52	4.57	4.47	4.44	4.41	4.32	4.35	4.22
Port 2 (GHz)	5.31	5.35	5.24	5.19	5.14	5.01	5.05	4.85
Port 3 (GHz)	6.00	6.05	5.91	5.85	5.79	5.63	5.68	5.44
Port 4 (GHz)	6.77	6.83	6.64	6.56	6.47	6.25	6.31	5.99

### Effect of positions of fluidic channels

**Figure 10:**
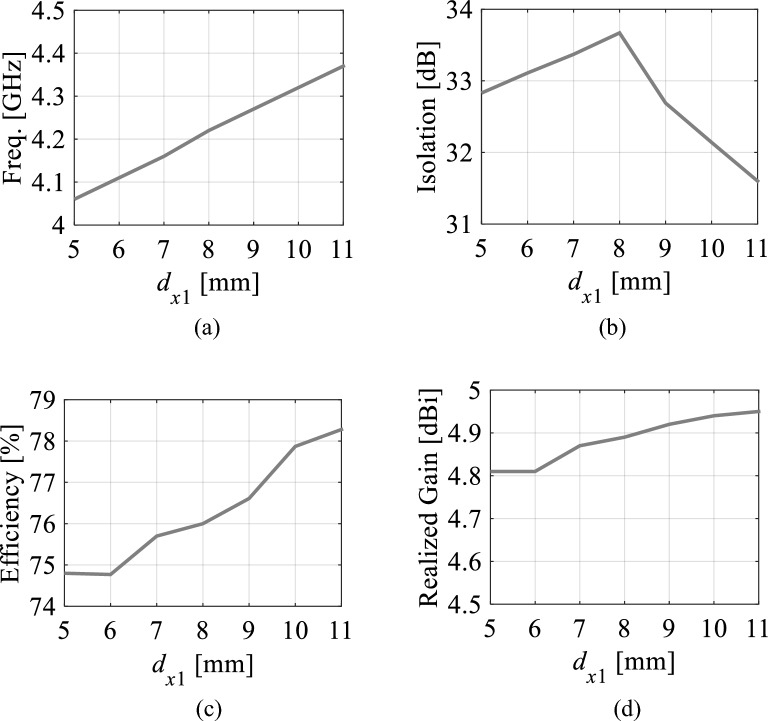
Antenna parameters at Port 1 with distilled water-filled fluidic channels as a function of *d*_*x*1_: (**a**) Operating frequency at Port 1, (**b**) isolation, (**c**) efficiency, and (**d**) realized gain.

**Figure 11:**
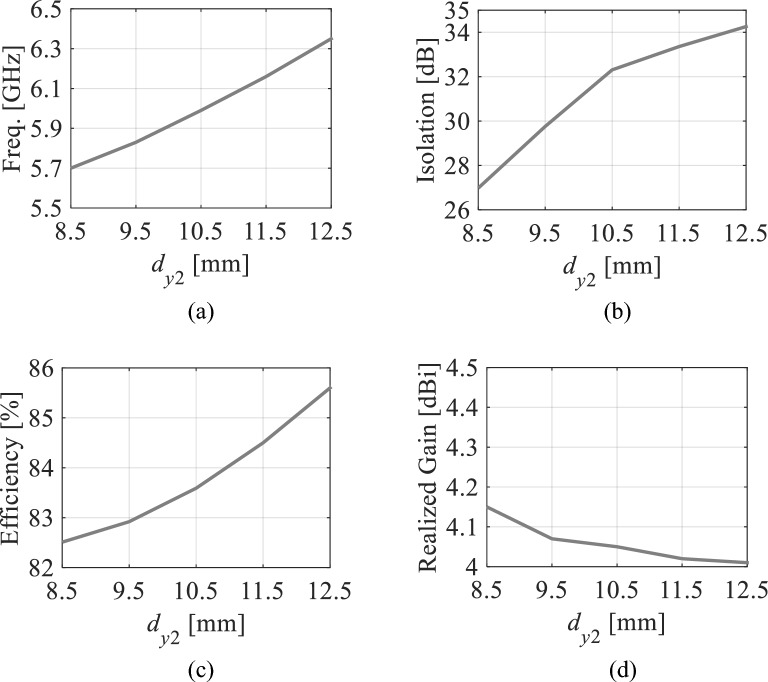
Antenna parameters at Port 4 with distilled water-filled fluidic channels as a function of *d*_*y*2_: (**a**) Operating frequency at Port 4, (**b**) isolation, (**c**) efficiency, and (**d**) realized gain.

The locations of the microfluidic channels are important for this antenna realization. It is observed that the distances *d*_*x*1_, *d*_*x*2_, *d*_*y*1_, and *d*_*y*2_ from the center *O* to the channels affect the antenna performances whereas the center-to-center distance *d*_4_ between the channels has no significant effect on the antenna performances. In order to explain this, we have carried out parametric analysis using the parameters *d*_*x*1_, *d*_*y*2_, and *d*_4_. Figures 10 and 11 represent the effect of positions of the channels on the antenna parameters (operating frequency, isolation, efficiency, and realized gain) associated with Ports 1 and 4, respectively. Referring to Fig. 10, the tuning range can be extended further as the operating frequency decreases by decreasing the *d*_*x*1_ but the antenna parameters such as isolation, efficiency, and realized gain also decrease. Therefore, the *d*_*x*1_ is chosen as 8 mm to achieve highest possible isolation associated at Port 1 with reasonable tuning range, efficiency, and realized gain. Similarly, as seen in Fig. 11, when the *d*_*y*2_ decreases the operating frequency decreases which will increase the tuning range but isolation and efficiency will also decrease. Hence the *d*_*y*2_ is chosen as 10.5 mm to achieve reasonable tuning range and antenna performances.

### Design guideline

Based on the above studies, a simple design approach can be formulated for realization of a frequency reconfigurable self-quadruplexing antenna.

*Step*-1: Design a SIW square cavity of dimension of 0.645*λ*_*g*_ × 0.645*λ*_*g*_ for operation in TE_110_ mode at 4.55 GHz and TE_210_/TE_120_ modes at 7.2 GHz.
*Step*-2: Realize Antenna-1 by employing four 50Ω feed lines to four sides of the square cavity.
*Step*-3: Produce four unequal quarter-mode cavity resonators with dimensions of 0.245*λ*_*g*_, 0.219*λ*_*g*_, 0.202*λ*_*g*_, and 0.21*λ*_*g*_ by inserting an X-shaped slot on the top plane of the cavity.
*Step*-4: Optimize the dimensions of each QMCRs to radiate at 4.5 GHz, 4.95 GHz, 5.33 GHz, and 6.75 GHz.
*Step*-5: Optimize the dimensions of inline feedings to achieve a good impedance matching between feed lines and QMCR resonators.
*Step*-6: Create two fluidic channels corresponding to each QMCR resonator with a diameter of 1.6 mm and height of 0.562 mm to enable frequency tunability to each band independently or simultaneously based on the specific requirements.
*Step*-7: To achieve reasonable tuning range and antenna performances, fix the locations of the fluidic channels at the distances of *d*_*x*1_ = 8 mm, *d*_*y*1_ = 10 mm, *d*_*x*2_ = 9 mm, and *d*_*y*2_ = 10.5 mm from center of the cavity associated with ports 1, 2, 3, and 4, respectively.
*Step*-8: Select *d*_4_ = 3 mm for easy fabrication, liquid filling, covering with copper tape, and measurements.
*Step*-9: Fill the fluidic channels with different dielectric liquids such as ethyl acetate, acetone, and distilled water to obtain resonant frequencies corresponding to various wireless standards/applications.
*Step*-10: Validate the tunable antenna through the EM simulation, prototyping, and measurements.

## Results

### Fabrication and measurement

**Figure 12:**
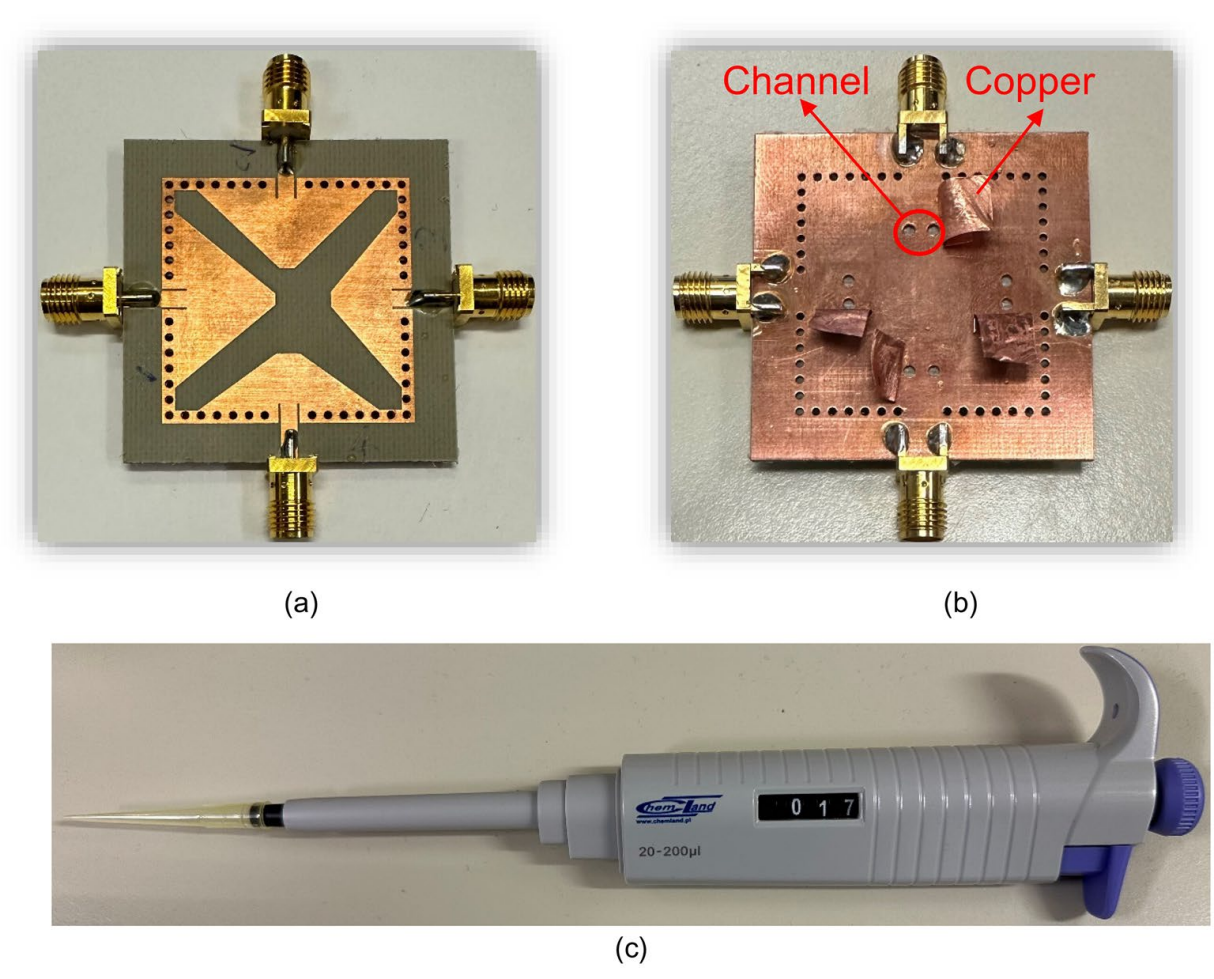
Photographs of the fabricated tunable antenna prototype: (**a**) front view, (**b**) back view, and (**c**) single-channel volume controlled pipette.

**Figure 13:**
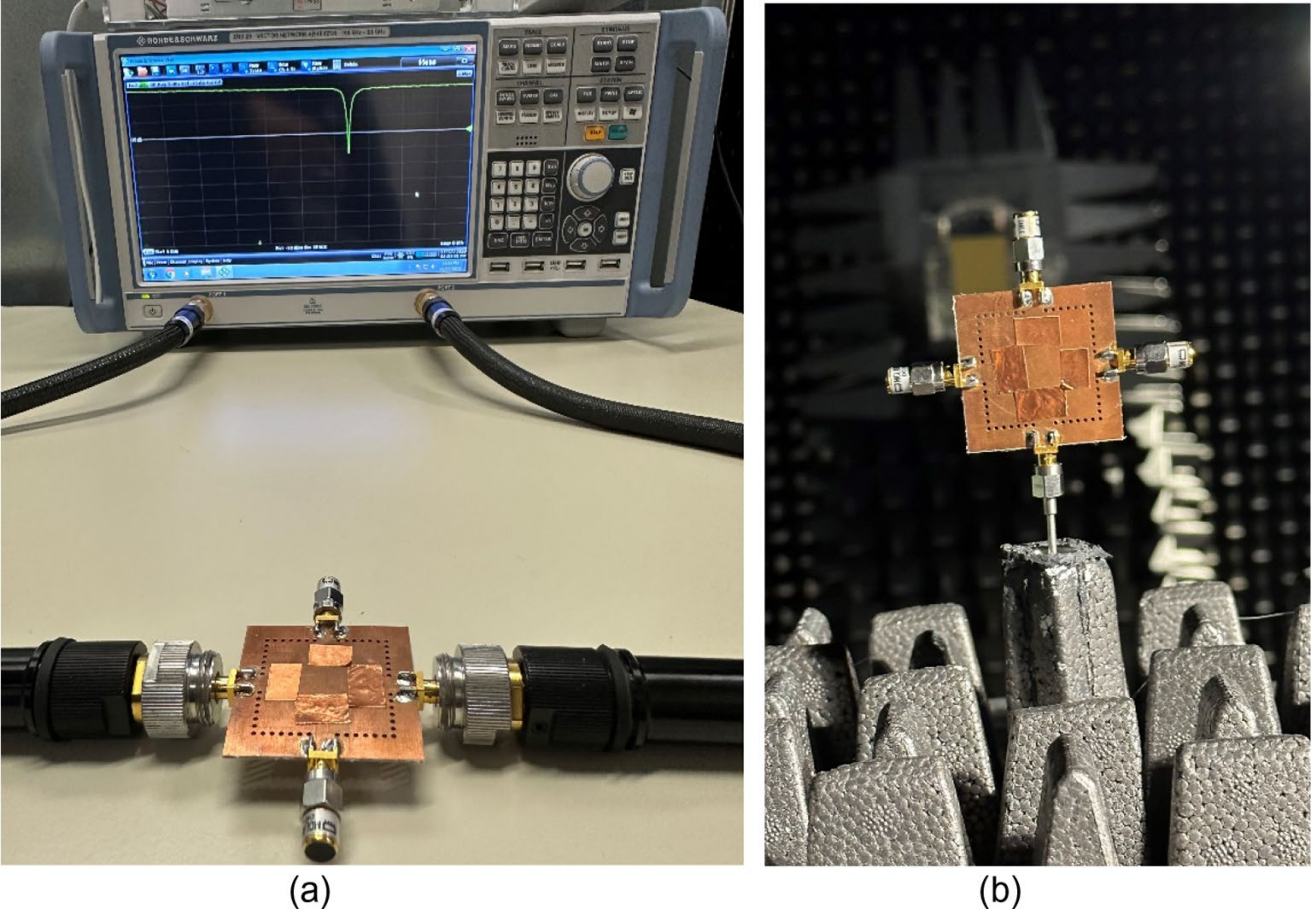
Measurement setup for the proposed tunable antenna prototype: (**a**) S-parameters, and (**b**) radiation performance.

For experimental verification, a microfluidically frequency reconfigurable self-quadruplexing antenna has been fabricated using Rogers AD250 PCB substrate (thickness of 0.762 mm, dielectric constant *ε*_*r*_ = 2.5, loss tangent *tanδ* = 0.0014). The fabricated tunable antenna has a footprint area of 0.37*λ*_*g*_, here *λ*_*g*_ is the guided wavelength at first resonating frequency. To facilitate tunability, two fluidic cylindrical channels are drilled from the bottom plane of each QMCR resonator without removing the metallization layer at the top plane and 0.2 mm dielectric substrate was left at the top to protect the radiating aperture. The diameter *d*_3_ and height *h*_*f*_ of each cylindrical channel are 1.6 mm and 0.562 mm, respectively. The front and back views of the prototype are shown in Fig. 12a and b, respectively. As seen in Fig. 12c, a single-channel volume controlled pipette is employed for injecting the fluid into the channels with the required amount of volume. After the respective channels are filled, the copper tape is place over the channels to restore the ground plane of the resonator as shown in Fig. 12b. Similarly, the retraction of the fluid is done by the pipette which has an air cushion used to create a vacuum and draw fluid into its chamber. Hence, different dielectric liquids can be employed to obtain the required operating frequencies. Figure 13 displays the measurement setup used for the fabricated tunable antenna. The S-parameters are measured using a Rodhe and Schwarz vector network analyzer as shown in Fig. 13a. The farfield features and radiation patterns have been measured using a fully automated anechoic chamber as depicted in Fig. 13b. The measurements are conducted with one port being activated and terminating the remaining ports with 50Ω loads. In order to experimentally demonstrate the frequency reconfigurability, distilled water and air are poured into the fluidic channels.

### S-parameters

**Figure 14:**
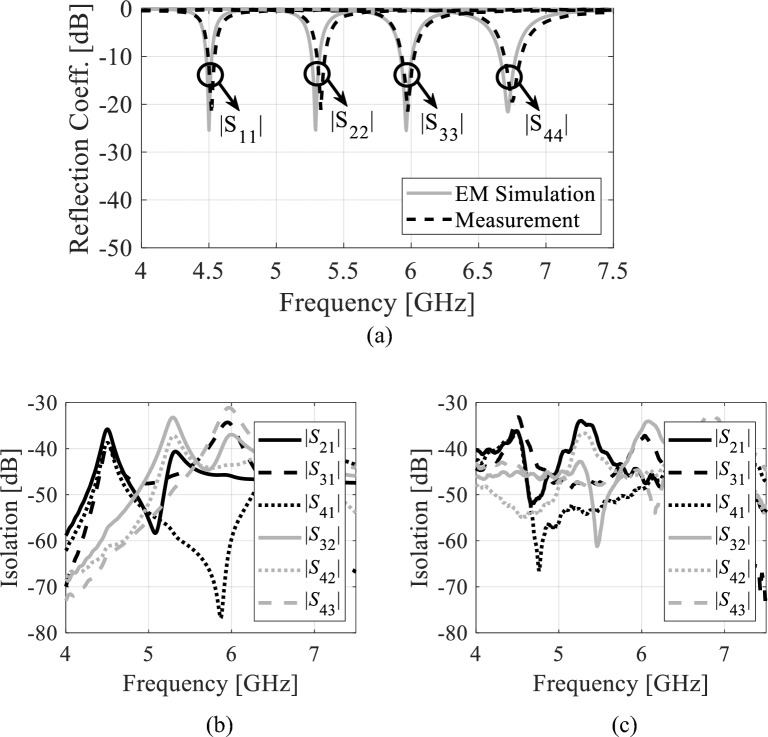
*S*-parameters of the antenna prototype without fluidic channels: (**a**) EM-simulated and measured reflection coefficients, (**b**) EM-simulated isolation, (**c**) measured isolation.

**Figure 15:**
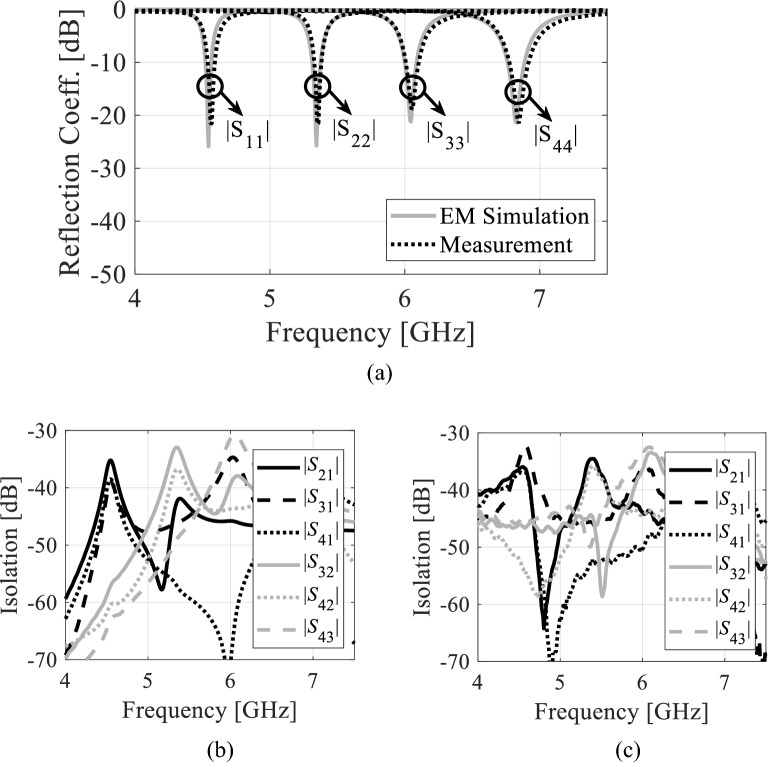
*S*-parameters of the tunable antenna prototype with air-filled fluidic channels: (**a**) EM-simulated and measured reflection coefficients, (**b**) EM-simulated isolation, (**c**) measured isolation.

**Figure 16:**
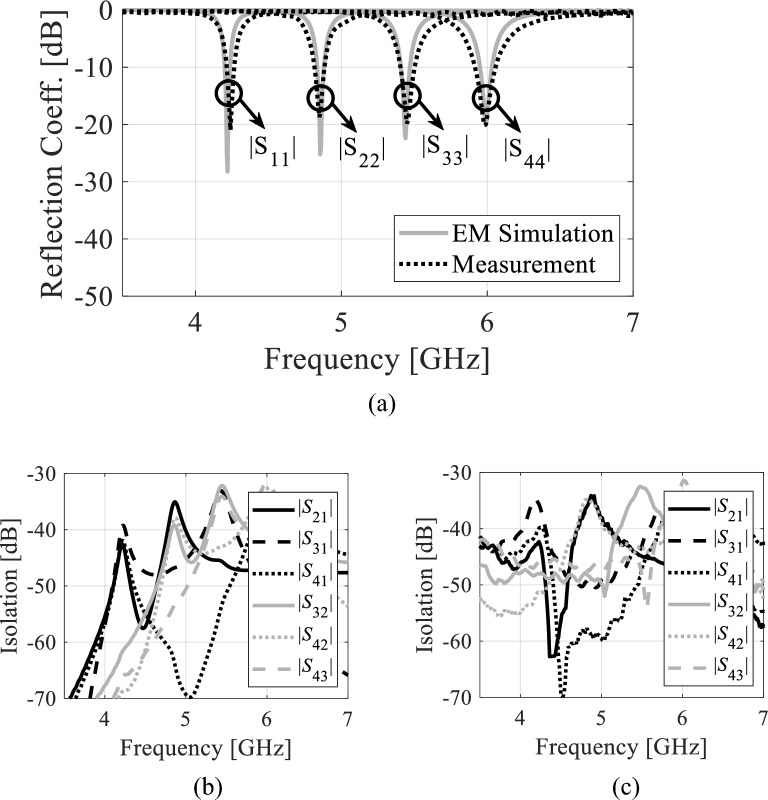
S-parameters of the tunable antenna prototype with distilled water-filled fluidic channels. (**a**) EM simulated and measured reflection coefficients, (**b**) EM simulated isolation, and (**c**) measured isolation.

Figure 14 illustrates the reflection and transmission coefficients of the proposed antenna without fluidic channels (i.e., channels filled with AD250 material). According to Fig. 14a, the measured reflection coefficients at 4.50 GHz, 5.33 GHz, 5.97 GHz, and 6.75 GHz are − 25.4 dB, − 25.37 dB, − 25.48 dB, and − 21.57 dB, respectively, whereas the EM-simulated reflection coefficients are − 20.12 dB, − 20.21 dB, − 20.23 dB, and − 19.32 dB. Referring to Fig. 14b and c, the measured isolations are greater than 33.23 dB whereas, the EM simulated isolations are better than 33.45 dB. Figure 15 demonstrates the S-parameters of the proposed tunable antenna with air-filled fluidic channels. This tunable antenna has measured passbands of 4.57 GHz, 5.35 GHz, 6.06 GHz, and 6.84 GHz. As seen in Fig. 15a, the reflection coefficients lower than − 19.21 dB are found in both EM simulations and measurements. From Fig. 15b and c, the measured and EM-simulated isolations are larger than 32.31 dB. Similarly, the measured and EM-simulated S-parameters of the prototype with distilled water-filled fluidic channels are illustrated in Fig. 16. This antenna radiates at 4.22 GHz, 4.85 GHz, 5.45 GHz, and 5.98 GHz. From Fig. 16a, it can be seen that the measured and EM-simulated reflection coefficients are below –18,84 dB at all frequency bands. Referring to Fig. 16b and c, the isolation of the tunable antenna with distilled water-filled fluidic channels are larger than 32.36 dB across all the frequency bands. The first, second, third, and fourth operating bands may be tuned to 350 MHz (8.3%), 510 MHz (10.5%), 610 MHz (11.2%), and 860 MHz (14.38%) overall using the proposed tunable self-quadruplexing antenna.

### Radiation patterns

**Figure 17:**
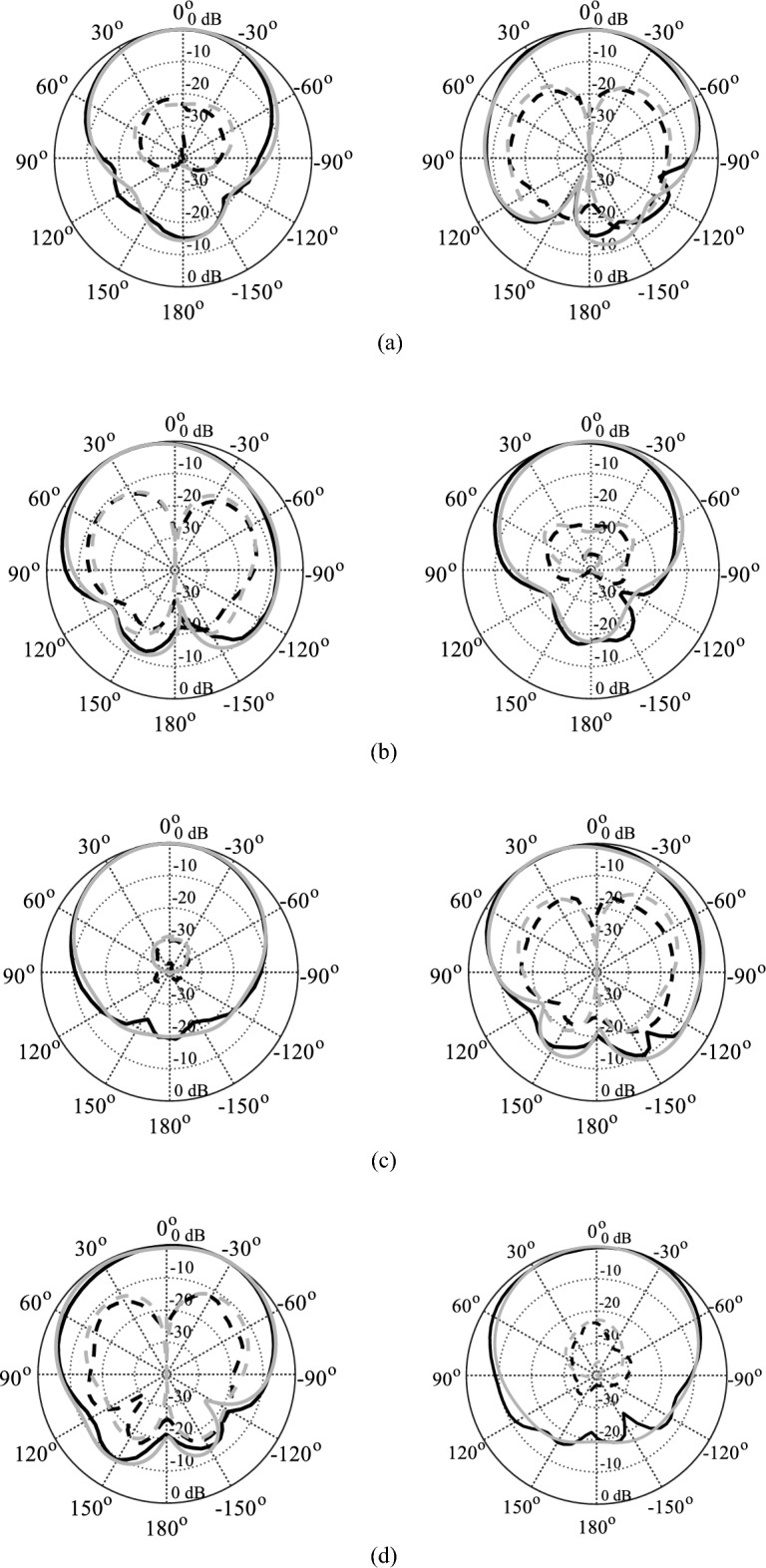
Normalized radiation pattern of the tunable antenna prototype with distilled water-filled fluidic channels: (**a**) 4.22 GHz, (**b**) 4.85 GHz, (**c**) 5.45 GHz, (**d**) 5.98 GHz [simulation**—**grey, measurement—black); H-plane (left), E-plane (right); copol—solid, crosspol—dashed].

**Figure 18:**
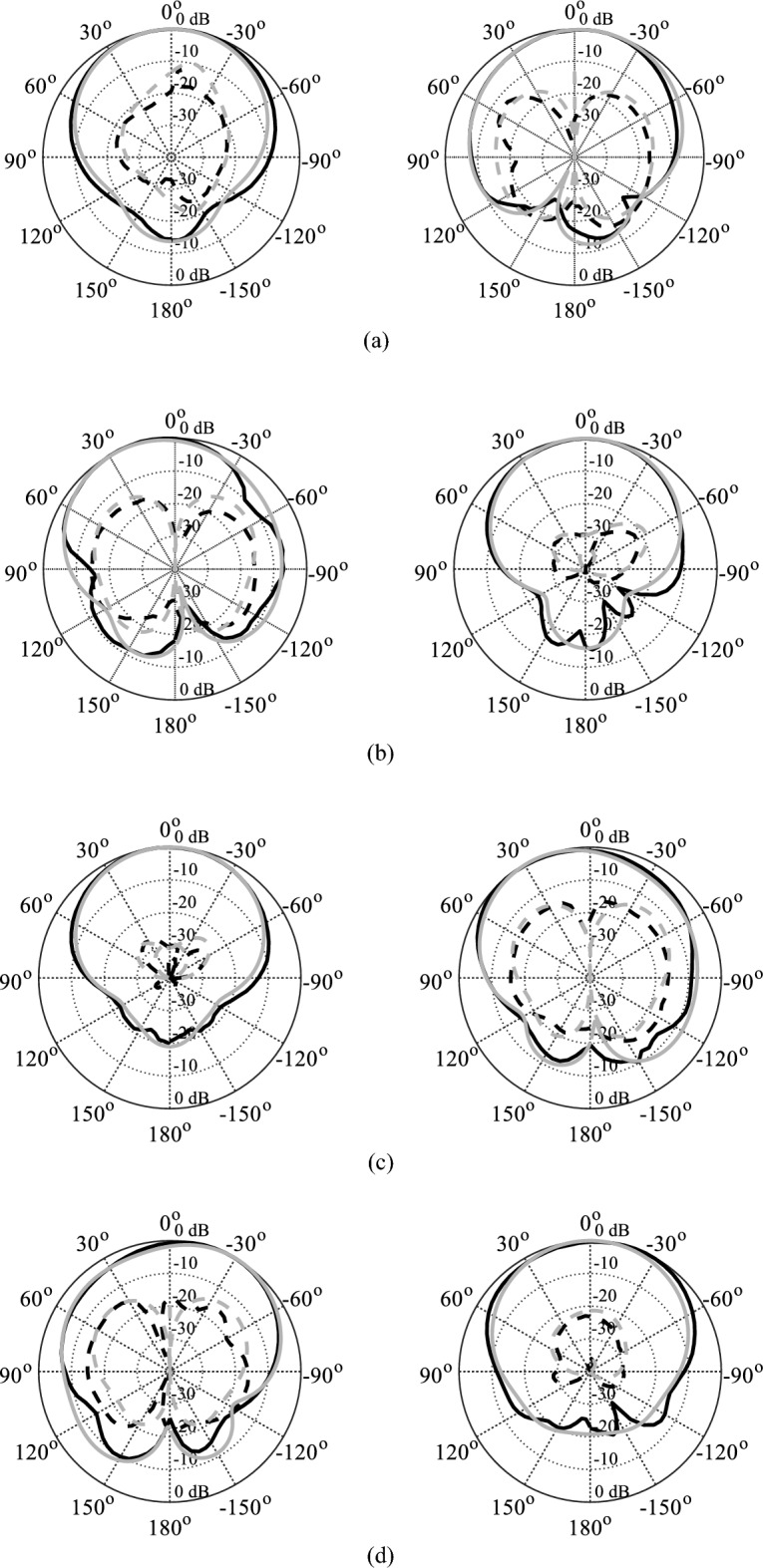
Normalized radiation pattern of the tunable antenna prototype with distilled water-filled fluidic channels: (**a**) 4.22 GHz, (**b**) 4.85 GHz, (**c**) 5.45 GHz, (**d**) 5.98 GHz [simulation—grey, measurement—black); H-plane (left), E-plane (right); copol—solid, crosspol—dashed].

The EM-simulated and measured radiation patterns of the tunable antenna prototype with air- and distilled water-filled fluidic channels are drawn in Figs. 17 and 18, respectively. For air-filled fluidic channels, the radiation patterns for excitation at Port 1 or 2 or 3 or 4 are illustrated in Fig. 17a–d, respectively.

A good agreement is observed between the measured and EM-simulated results at all frequency bands. The radiation patterns are measured at E-plane (ϕ = 0°) and H-plane (ϕ = 90°) for different port excitation. Both measured and EM-simulated radiation patterns are stable and unidirectional in the E- and H-planes at all frequency bands. It can be seen that the EM-simulated cross-polarization level is less than − 22.32 dB, whereas the measured cross-polarization level is less than − 22.01 dB at all frequency bands. The measured and EM-simulated front-to-back ratio (FTBR) is better than 15.80 dB at all operating bands. Figure 18a–d show radiation patterns for excitation at Port 1 or 2 or 3 or 4 in distilled water-filled fluidic channels. As seen in Fig. 18, the measured cross-polarization level and FTBR are better than − 20.16 dB and 15.02 dB, respectively.

### Realized gain

**Figure 19:**
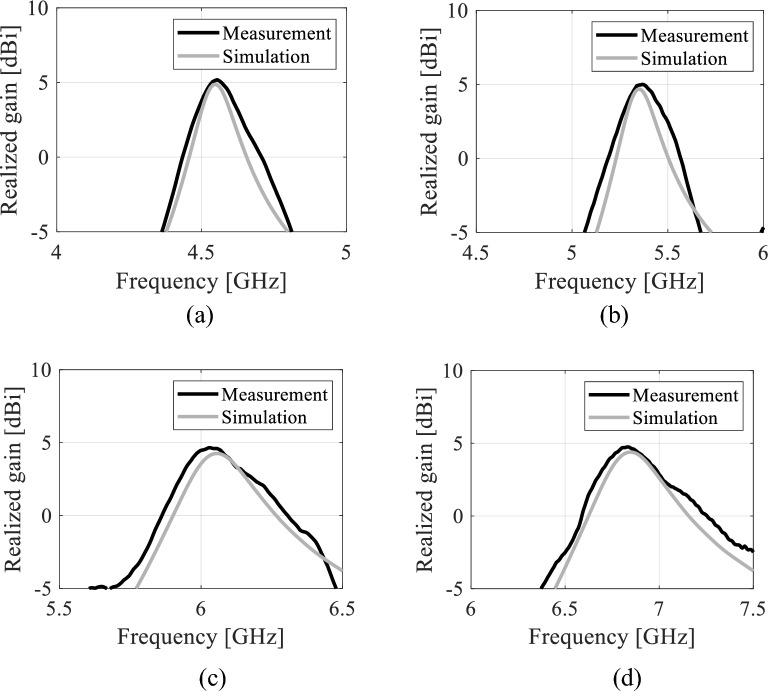
Realized gain of the tunable antenna prototype with air-filled fluidic channels: (**a**) 4.57 GHz, (**b**) 5.35 GHz, (**c**) 6.06 GHz, (**d**) 6.84 GHz.

**Figure 20:**
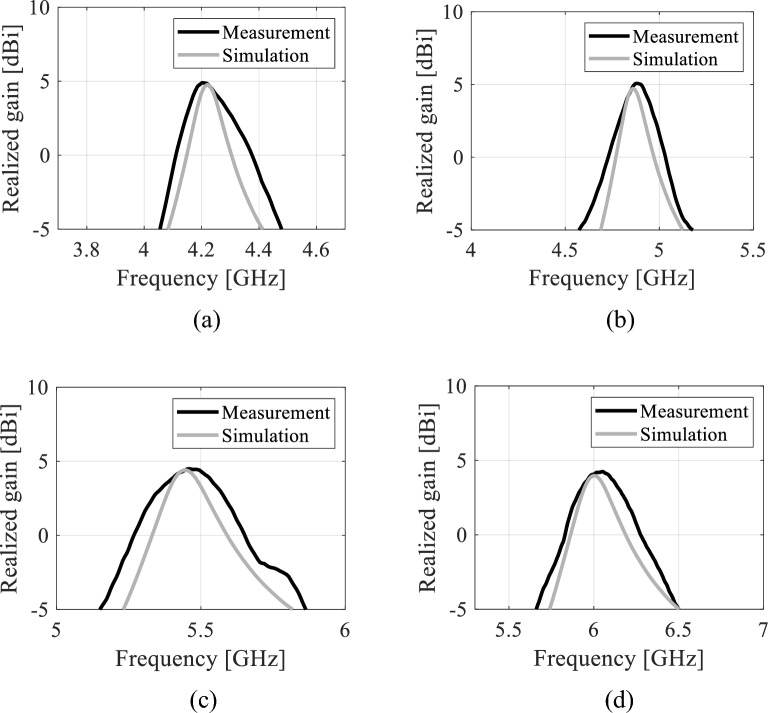
Realized gain of the tunable antenna prototype with distilled water-filled fluidic channels: (**a**) 4.22 GHz, (**b**) 4.85 GHz, (**c**) 5.45 GHz, (**d**) 5.98 GHz.

Figures 19 and 20 show the realized gain of the proposed tunable antenna with air- and distilled water-filled fluidic channels, respectively. The EM simulations and the measured realized gains are in good agreement. Minor variations can be attributed to dielectric loss, manufacturing imperfections, connector soldering, and measurement tolerance. However, the error does not exceed five percent. As seen in Fig. 19, the EM-simulated minimum realized gain is greater than 4.45 dBi while the measured minimum realized gain is 4.55 dBi at all frequency bands. Table 3 shows the measurement performance of the tunable antenna with air-filled fluidic channels. For, distilled water-filled fluidic channels, the measured and EM-simulated realized gains at all frequency bands are 4.05 dBi and 4.15 dBi, respectively. Table 4 presents the measured antenna performance with distilled water poured into the fluidic channels. Table 5 provides a summary of the proposed tunable antenna prototype's reconfigurable performances.

**Table 3:** Measured tunable antenna parameters for air-filled channels.

Port excitation	Tunable freq. (GHz)	Minimum isolation (dB)	Realized gain (dBi)	Cross polarization (dB)	FTBR (dB)
Port 1	4.57	32.31	5.05	23.24	15.8
Port 2	5.36	34.66	4.96	26.24	17.71
Port 3	6.06	32.59	4.55	22.09	20.58
Port 4	6.84	36.56	4.71	24.81	21.32

**Table 4:** Measured tunable antenna parameters for distilled water-filled channels.

Port excitation	Tunable freq. (GHz)	Minimum isolation (dB)	Realized gain (dBi)	Cross polarization (dB)	FTBR (dB)
Port 1	4.22	35.43	4.85	20.16	15.02
Port 2	4.85	34.89	4.82	28.88	18.42
Port 3	5.45	32.94	4.40	22.02	19.60
Port 4	5.98	32.36	4.05	20.25	22.58

**Table 5:** Measured performances of the proposed tunable antenna.

Port excitation	Tunable freq. (GHz)	Minimum isolation (dB)	Realized gain (dBi)
Port 1	4.22–4.57	35.43–32.31	4.85–5.05
Port 2	4.85–5.36	34.89–34.66	4.82–4.96
Port 3	5.45–6.06	32.94–32.59	4.40–4.55
Port 4	5.98–6.84	32.36–36.56	4.05–4.71

### Comparative analysis

The performance of the proposed frequency-tunable self-quadruplexing antenna (SQA) is compared with that of previously reported SQAs^16–24^, and summarized in Table 6. The proposed SQA offers frequency reconfigurability using fluidic channels, which has been demonstrated for the first time in the literature, and not realized in the reported SQAs^16–24^. The proposed frequency-tunable SQA shows the highest isolation compared to the published SQAs in^16–24^, with the exception of the SQA in^23^. Compared to the reported SQAs^16–24^, the proposed tunable antenna offers reasonable realized gain, FTBR, and cross-polarization level. The footprint area of our SQA is less than that of^16,17,24^. Furthermore, an equivalent circuit model was implemented to confirm and explain the operation of the suggested frequency-adjustable antenna. On the contrary, no equivalent network model was provided for SQAs presented in^16–19,22^.

**Table 6:** Performances comparison between proposed tunable SQA and reported SQAS.

Ref	Frequency band (GHz)	ISL (dB)	Realized gain (dBi)	Size (λ_g_^2^)	Equivalent circuit model	Self-quadruplexing	Reconfigurable
^16^	8.19/8.8/9.71/11	> 22	5.5/6.9/7.47/7.45	0.792	No	Yes	No
^17^	8.85/10.4/11.4/12.25	> 27	5.2/6/6.25/7	1.369	No	Yes	No
^18^	3.5/4.9/5.4/5.8	> 23.1	4.4/5.07/5.4/5.7	0.32	No	Yes	No
^19^	5.14/5.78/6.74/7.74	> 28	4.1/4.96/6.2/6.1	0.27	No	Yes	No
^20^	2.45/3.5/4.9/5.4	> 29.9	3.85/5.33/5.95/5.97	0.23	Yes	Yes	No
^21^	5.8/7.4/28/38	> 26	4.1/5.2/6.1/8.3	0.25	Yes	Yes	No
^22^	4.67–5.84	> 16	0.4 to 3.73	0.211	No	Yes	No
^23^	2.33/2.96/5.43/6.15	> 32.5	4.31/3.39/6.12/4.43	0.124	Yes	Yes	No
^24^	4.8/5.4/28/30	> 20	5.4/5.2/8/8.7	0.51	Yes	Yes	No
Proposed	4.22–4.57/4.85–5.36/5.45–6.06/5.98–6.84	> 32.31	4.85–5.05/4.82–4.96/4.40–4.55/4.05–4.71	0.37	Yes	Yes	Yes

ISL: isolation, λ_g_: guided wavelength at lower frequency band.

## Conclusion

In this article, a novel microfluidically frequency reconfigurable self-quadruplexing antenna has been developed and demonstrated. The proposed antenna has been implemented using a substrate-integrated waveguide square cavity, four 50Ω feed lines, an X-shaped slot, and fluidic channels. Four quarter-mode cavity resonators have been created by engraving the X-shaped slot on the top plane of the cavity to produce four resonating frequency bands. The inline feeding technique has been employed in order to obtain good impedance matching between the feed lines and the resonators. Each quarter-mode cavity resonator has two fluidic channels that allow for frequency reconfigurability. Different dielectric liquids with high relative permittivity may be used to adjust the resonating frequencies independently or concurrently. Multiple scenarios are possible with fluidic channels, providing flexibility in frequency tunability according to the system requirements. The tunability has been investigated by filling the fluidic channels with air, ethyl acetate, acetone, and distilled water. A simple design approach has been provided for the realization of the antenna. Furthermore, an equivalent circuit has been generated to verify the operating principles of the proposed antenna. Finally, a microfluidically frequency-tunable self-quadruplexing antenna has been manufactured and tested. For experimental demonstration, the fluidic channels are filled with air or distilled water. For the first, second, third, and fourth operating bands, the antenna shows frequency tuning ranges of 350 MHz (8.3%), 500 MHz (10.3%), 610 MHz (11.2%), and 845 MHz (14.1%), respectively. The SQA prototype offers minimum isolation and realized gain ranges from 32.31 to 36.56 dB, and from 4.05 to 5.05 dBi, respectively. Consequently, the proposed frequency tunable self-quadruplexing antenna is the first in its category suitable for several communication systems. Since, this is a fully passive frequency tuning method, an auto-fill technique of dielectric liquids can be explored for the realization for frequency tuning self-quadruplexing antennas.

## Data Availability

The datasets used and/or analyzed during the current study available from the corresponding author on reasonable request.
